# Assessment of patient information quality provided by artificial intelligence-based large language models on cryptorchidism: a comparison of ChatGPT-5, Gemini, and Grok

**DOI:** 10.1590/1806-9282.20260541

**Published:** 2026-08-28

**Authors:** Doruk Demirel, Kazım Ceviz, Tanju Keten, Cevdet Serkan Gökkaya

**Affiliations:** 1 Ankara Bilkent City Hospital, Department of Urology – Ankara, Türkiye.

**Keywords:** Cryptorchidism, Artificial intelligence, Large language models, Patient education as topic, Health literacy

## Abstract

**OBJECTIVE:**

The aim of this study was to evaluate the responses generated by ChatGPT-5, Gemini, and Grok to the “six most frequently asked patient questions” on cryptorchidism published by the European Association of Urology, assessing them in terms of quality, understandability, actionability, and readability.

**METHODS:**

ChatGPT-5, Gemini, and Grok were asked these six frequently asked questions listed on the European Association of Urology patient information page on cryptorchidism. The quality of the responses was evaluated using the Quality Assessment Tool for Patient Health Information instrument, understandability and actionability were assessed using Patient Education Materials Assessment Tool scores, and readability was measured with the Coleman-Liau Index. All evaluations were performed by four urologists.

**RESULTS:**

Among the three artificial intelligence-based large language models, Gemini achieved the highest mean Quality Assessment Tool for Patient Health Information and Patient Education Materials Assessment Tool for Printable Materials scores. Kruskal-Wallis analysis demonstrated a statistically significant difference in Quality Assessment Tool for Patient Health Information scores among the groups (p=0.005); pairwise comparisons revealed that Gemini scored significantly higher than ChatGPT-5 (p=0.001). No significant differences were observed among the models for Patient Education Materials Assessment Tool—Understandability Section or Patient Education Materials Assessment Tool—Actionability Section scores (p>0.05). In the readability analysis, Grok had the highest Coleman-Liau Index value (Coleman-Liau Index=14.68), and all models produced texts requiring a university-level reading ability. Although the Gemini model achieved higher overall quality scores, all artificial intelligence-based large language models provided good-quality but difficult-to-read information.

**CONCLUSION:**

The responses generated by all three models demonstrated high levels of understandability and strong actionability. We anticipate that future, more advanced versions of artificial intelligence-based large language models will further improve these outcomes and contribute positively to the existing literature.

## INTRODUCTION

Cryptorchidism is a common urological issue found in newborn boys^1^. It may be present on one or both sides, and its incidence among term infants is reported to range from 1 to 5%^2^. It is known that this condition elevates the risk of fertility disorders and testicular cancer in adulthood^3^. So, early detection of cryptorchidism and surgical management at the right time is necessary. This places considerable responsibility on parents, as seeking medical care depends largely on their ability to access clear, reliable, and easy-to-understand information about the condition.

In recent years, artificial intelligence-based large language models (AI-LLMs) have contributed significantly to the dissemination and accessibility of medical information^4^. In a study conducted in 2023 with the participation of 456 urologists, half of the respondents reported using AI-LLMs in their clinical practice^5^. As the use of these models continues to expand, one of the major concerns is the risk of producing incomplete or inaccurate information due to their limited access to up-to-date and reliable sources^6^. Therefore, evaluating the reliability, quality, and comprehensibility of the responses provided by AI-LLMs is of great importance^7^. In the literature, the performance of AI-LLMs has been examined in various topics such as urolithiasis and premature ejaculation, and notable findings have been reported in these areas^8,9^. However, to date, there is no study in the current literature that evaluates the response quality of AI-LLMs specifically on cryptorchidism.

In this study, the six “most frequently asked questions by patients” published on the European Association of Urology (EAU) patient information page concerning cryptorchidism were posed to three AI-LLMs—ChatGPT-5, Gemini, and Grok. The responses were then assessed in terms of quality, understandability, actionability, and readability.

## METHODS

Since this study was conducted using publicly accessible AI-LLMs, ethical approval was not required. Google Chrome (Version 141.0.7390.67, arm64) was used in incognito mode, and all prior browsing data were cleared. The six “most frequently asked questions by patients” listed on the EAU patient information page on cryptorchidism were submitted to three different AI-LLMs (ChatGPT-5, Gemini, and Grok)^10^.

Each question was asked once, and all responses were recorded. The answers were independently evaluated by four urologists using the Quality Assessment Tool for Patient Health Information (DISCERN), the Patient Education Materials Assessment Tool—Understandability section (PEMAT-PU), the Patient Education Materials Assessment Tool—Actionability section (PEMAT-PA), and the Coleman-Liau Index (CLI). To minimize potential bias arising from brand recognition, all evaluators were blinded to the source AI model during the assessment process; the responses were anonymized and presented in a randomized order prior to evaluation.

These instruments were selected based on their widespread validation and established use in evaluating AI-generated patient health information in the urology literature^11,12,13,14^. DISCERN was chosen for its ability to assess the reliability and quality of written health information across multiple dimensions^15^. PEMAT-PU and PEMAT-PA were selected because they are specifically designed to evaluate the understandability and actionability of patient education materials, including those produced in digital or AI-based formats^16^. The CLI was employed as a standardized, objective measure of text readability that does not require manual scoring. Collectively, these tools allow a comprehensive, multi-dimensional assessment of AI-generated patient information^17^.

DISCERN is a 16-item instrument used to assess the reliability and quality of health information. Each DISCERN item is rated on a 5-point Likert scale ranging from 1=poor to 5=excellent. In accordance with previous studies, mean item scores (range: 1–5) were used for comparison. Accordingly, scores of 4.0–5.0 were considered excellent, 3.0–3.9 good, 2.0–2.9 fair, 1.5–1.9 poor, and 1.0–1.4 very poor. PEMAT-PU measures the understandability of the material, whereas PEMAT-PA assesses its actionability or applicability. PEMAT-PA consists of 7 items and PEMAT-PU of 19 items; both are scaled from 0 to 100. For all three scales, the mean of the scores provided by the two urologists was used.

In addition, the readability levels of the generated texts were analyzed using the CLI. This index estimates the grade level (based on the US education system) required to understand the text by calculating the average number of letters per 100 words and the average number of sentences per 100 words. Higher CLI values indicate lower readability. A score of 11 or higher corresponds to a reading level requiring at least college-level proficiency. Using this index, the readability levels of the responses generated by all three models were determined.

### Statistical analysis

All analyses were performed using IBM SPSS Statistics software (version 2024; IBM Corp., Armonk, NY, USA). Data were summarized using descriptive statistics and presented as mean±standard deviation along with minimum–maximum values. The normality of distribution was assessed using the Shapiro-Wilk test. For variables that did not meet the assumption of normality, nonparametric tests were employed.

To evaluate whether the responses to the same questions differed among the three AI-based large language models, the Kruskal-Wallis test was applied. For variables in which significant differences were identified, pairwise comparisons were performed using the Mann-Whitney U test with Bonferroni correction. The CLI score was calculated using the standard formula based on the number of letters and sentences per 100 words. A p-value of <0.05 was considered statistically significant.

## RESULTS

Gemini achieved the highest average scores in DISCERN and PEMAT-P among the three AI-LLMs (Table 1). Kruskal-Wallis analysis revealed a statistically significant difference in DISCERN scores among the ChatGPT-5, Gemini, and Grok groups (χ^2^=10.426, p=0.005). According to the mean ranking scores, Gemini was ranked highest, followed closely by Grok and ChatGPT-5 (Mean rank: 162.33, 146.02, 125.15) (Table 1).

**Table 1:** Summary of Quality Assessment Tool for Patient Health Information and Patient Education Materials Assessment Tool-based quality, understandability, and actionability scores of patient education materials generated by artificial intelligence-based large language models.

Score type	ChatGPT-5 mean±SD	Gemini mean±SD	Grok mean±SD	χ^2^ (df)	p	Post-hoc results
DISCERN	3.09±1.50	3.72±1.62	3.41±1.63	10.426	0.005	G>C (0.001); Gr>C (0.092); G vs. Gr (0.183)
PEMAT-PU	86.9±5.2	92.7±4.3	92.9±4.7	4.866	0.088	G>C (0.056); Gr>C (0.060); G vs. Gr (1.000)
PEMAT-PA	86.7±10.3	96.7±8.2	90.0±16.7	2.583	0.275	G>C (0.093); Gr>C (0.476); G vs. Gr (0.461)

DISCERN Instrument: Quality Assessment Tool for Patient Health Information, PEMAT-PU: Patient Education Materials Assessment Tool—Understandability Section, PEMAT-PA: Patient Education Materials Assessment Tool—Actionability Section. C: ChatGPT-5; G: Gemini; Gr: Grok.

In pairwise comparisons, Gemini yielded significantly higher DISCERN scores compared with ChatGPT-5 (Z=-3.319, p=0.001). No significant differences were observed between the Grok–ChatGPT-5 and Grok–Gemini comparisons (p>0.05) (Table 1).

For PEMAT-PU and PEMAT-PA scores, no statistically significant differences were detected among the three groups (p>0.05). Although Gemini achieved the highest mean ranks in both sections, these differences were not statistically significant (p>0.05) (Table 1).

The readability of the texts generated by the three AI-based large language models was evaluated using the CLI. According to the analysis, Grok exhibited the highest readability index (CLI=14.68), while Gemini (CLI=13.39) and GPT (CLI=13.21) showed very similar values (Table 2).

**Table 2:** Readability parameters and Coleman-Liau Indices of texts produced by three artificial intelligence-based large language models.

Chatbot	Letters	Words	Sentences	L (letters/100 words)	S (sentences/100 words)	CLI
Grok	29,838	5,511	253	541.43	4.59	14.68
Gemini	11,454	2,217	89	516.64	4.01	13.39
GPT	8,551	1,661	71	514.81	4.27	13.21

CLI: Coleman-Liau Index.

## DISCUSSION

This study represents the first comparative analysis in the literature evaluating the quality, understandability, actionability, and readability of information generated by AI-LLMs on the topic of cryptorchidism. The findings show that although the models provided highly understandable and actionable content, the readability levels generally required a university-level reading ability. This indicates that AI-LLMs hold promising potential for patient education, but improvements are still needed in terms of linguistic simplicity and source reliability.

In a study assessing the quality of information provided by four different AI-LLMs on hypospadias, the median DISCERN score was reported as 4, indicating high-quality content^18^. Likewise, Alshak et al.^19^ found that ChatGPT produced high-quality responses in the field of reconstructive urology, with a mean DISCERN score of 3.63. Gibson et al. demonstrated that ChatGPT provided higher-quality information on prostate cancer compared with traditional non–AI-based informational websites^13^. In the current study, Gemini achieved the highest DISCERN score (3.72), whereas ChatGPT-5 had the lowest (3.09). These findings suggest that all three AI-LLMs produced good-quality responses for cryptorchidism, consistent with previous literature.

Gonultas et al.^9^ reported that ChatGPT, Gemini, and other conversational models generally provided moderate-to-high understandability when answering questions about premature ejaculation (PEMAT-P scores: Copilot 70, Gemini 72, ChatGPT-4o 83, ChatGPT-4o Plus 77, DeepSeek 79). However, in a study by Erkan et al.^20^ evaluating responses of AI-LLMs regarding treatment decisions for patients with urogenital cancers, the understandability scores (PEMAT-PU) were reported to be low. In contrast, the present study demonstrated high understandability scores across all three models (ChatGPT-5: 86.9, Gemini: 92.7, Grok: 92.9). This suggests that AI-LLMs use clearer and more comprehensible language when generating content on cryptorchidism. Notably, Gemini and Grok provided more consistent results in terms of fluency and sentence structure compared with ChatGPT-5.

Actionability (PEMAT-PA) values are typically reported around 40% in the literature, whereas the scores in this study ranged between 86 and 96%^18,19,21,22^. This marked increase may be attributed to the direct, step-by-step, and actionable nature of the cryptorchidism-related content being evaluated. Another contributing factor may be that parents are generally highly motivated to take immediate action regarding their child’s health. The high actionability scores observed in this study suggest that modern AI-LLMs are not only capable of providing information but also of producing content that helps guide patients toward appropriate actions.

Readability is often an important criterion for evaluating AI-LLMs. Stapleton et al.^21^, in their study assessing the responses of various AI-LLMs (ChatGPT-3.5, Perplexity, ChatSonic, and Bing AI) to frequently asked questions about Wilms tumor, reported that the produced texts required at least high school or university-level reading skills. Similarly, another study evaluating four AI-LLMs (ChatGPT-4.0, Perplexity, ChatSonic, and Bing AI) regarding interstitial cystitis also found that readability levels required at least a university-level education, which the authors highlighted as a limiting factor^22^. In the present study, the readability of the responses generated by three AI-LLMs was evaluated using the CLI. Grok had the highest readability score, whereas the other models demonstrated similar values. All three AI-LLMs required at least a university-level reading ability, which may limit comprehension among parents or patients with lower educational backgrounds.

An important consideration specific to cryptorchidism is the high level of parental anxiety associated with this diagnosis. Unlike adult patients seeking information on urological conditions, the primary recipients of AI-generated information in cryptorchidism are parents of newly diagnosed infants or young boys^23^. This demographic is often emotionally distressed, facing concerns about their child’s future fertility and cancer risk, and seeking immediate, actionable guidance. In this context, the high actionability scores observed in this study are particularly encouraging, as they suggest that AI-LLMs can effectively guide anxious parents toward appropriate next steps. However, the university-level readability requirements identified across all three models represent a meaningful barrier for parents with lower educational backgrounds. Future AI-LLM iterations should prioritize plain-language output tailored to this demographic, potentially offering adjustable readability levels to accommodate varying levels of health literacy. Integrating empathetic tone and reassurance into AI-generated responses may also help mitigate parental anxiety while ensuring accuracy and clinical relevance.

This study has several limitations. First, it is limited to the six standardized questions from the EAU patient information page; therefore, the findings may not be generalizable to more complex or case-specific questions. Second, to avoid influencing the models’ response patterns, each question was posed only once. Although methodologically appropriate, this approach may not fully reflect the variability of responses that might occur in real-world usage. Finally, all evaluated platforms are continuously updated and retrained, meaning that the reproducibility of the study may vary over time.

In conclusion, although Gemini achieved the highest overall quality scores, all three AI-LLMs produced good-quality yet difficult-to-read information. The responses from all models demonstrated high understandability and strong actionability. We believe that future versions of AI-LLMs will further improve and positively influence findings in the existing literature.

## Data Availability

The datasets generated and/or analyzed during the current study are available from the corresponding author upon reasonable request.
